# Distinguishing Tuberculosis and Crohn's Disease in Developing Countries: How Certain Can You Be of the Diagnosis?

**DOI:** 10.4103/1319-3767.49012

**Published:** 2009-04

**Authors:** Udayakumar Navaneethan, Jijo V. Cherian, Rajesh Prabhu, Jayanthi Venkataraman

**Affiliations:** Department of Internal Medicine, University of Cincinnati College of Medicine, Cincinnati, Ohio 45267 USA; 1 Department of Gastroenterology, Stanley Medical College, Chennai-600 001, India

**Keywords:** Crohn's disease, granulomatous colitis, endemic, inappropriate, tuberculosis

## Abstract

Distinguishing tuberculosis and Crohn's disease in patients presenting with chronic abdominal pain and diarrhea is a huge diagnostic challenge, particularly in tuberculosis endemic countries. A large number of patients with Crohn's disease are initially misclassified as having Intestinal tuberculosis in places where tuberculosis is endemic before they are treated for Crohn's disease. Although a variety of endoscopic, radiological and histological criteria have been recommended for the differentiation, it often proves difficult in routine clinical practice. Future prospective studies are required in patients with granulomatous colitis to prevent unnecessary inappropriate anti tuberculous therapy for patients with Crohn's disease and appropriate early treatment for a patient with tuberculosis.

Patients presenting with chronic abdominal pain and diarrhea who undergo endoscopic biopsies and have granulomatous colitis without casseation necrosis are a huge diagnostic challenge. This clinical situation epitomizes the challenge physicians in endemic countries face, wherein both intestinal tuberculosis (TB) and Crohn's disease (CD) coexist. Despite major advances in investigative tools, including molecular studies, the differential diagnosis of these two conditions poses a great challenge to clinicians' worldwide.[1]

Why is it important to differentiate these two conditions? The answer is simple. The ultimate course of these two disorders is very different. Along with an increase in the incidence of TB, there has been a proportionate increase in the incidence of intestinal TB.[2,3] Whereas TB is an entirely curable disease, CD, in contrast, is a progressive and relapsing illness. While most CDs respond to mesalamine preparations, immunotherapy or steroid treatment, a small proportion even respond to antituberculous therapy (ATT), making the issue even more confusing. Conversely, steroid therapy will do more harm than good in individuals with intestinal TB. CD also requires life-long treatment and follow-up. Thus, differentiating between the two diseases is very important. Unfortunately, it is difficult to differentiate intestinal TB from CD because of similar clinical, pathological, radiological and endoscopic findings. Although attempts have been made to distinguish them, there are still no specific features to differentiate them.

Although we tend to think that physicians in developing countries where TB is common face the brunt of the problem, the problem is global. With the reemergence of TB in the West in the wake of the acquired immunodeficiency syndrome epidemic,[2,4,5] the ability to cure TB with appropriate antibiotic treatment and the emergence of CD in many tropical countries where it was previously unknown and histological differentiation of these two disorders assumes an even greater importance than ever before.

In fact, a large number of patients with CD are initially misclassified as having intestinal TB in places where TB is endemic before they are treated for CD because of failure to improve with ATT. Where appropriate, patients with an uncertain diagnosis are usually given ATT and a final diagnosis is made on the basis of response to the treatment. There are high rates of misdiagnosis in both conditions. For example, 65% of CD had been misdiagnosed as TB, as reported by Tonghua ***et al.*** from China.[6]

For this reason, many investigators have attempted to find specific differential diagnostic methods to distinguish these conditions. A variety of clinical, endoscopic and radiological criteria have been recommended for the differentiation, [7–12] but these criteria have been demonstrated to have their limitations too. A contrast-enhanced computed tomography scan shows prominent pericolic or perienteric vasculature - increased number, tortuosity and widely spaced vasa recta of the ileum, referred to as “vascular jejunization of the ileum” or the “comb sign.” Abrupt tapering, right angle branching, early, dense venous opacification, the comb sign and increased opacification of the bowel wall are all suggestive of active CD.[13] In TB, the ileocaecal and adjacent medial wall of the caecum appears asymmetrically thickened, with advanced lesions showing gross wall thickening, adherent loops, large regional nodes and mesenteric thickening that form a soft tissue mass. Presence of ascites and caseating lymph nodes suggest abdominal TB rather than CD. (Personal observations, V.J.)

On colonoscopy, colonic TB may present as an inflammatory stricture, hypertrophic lesions resembling polyps or tumors, segmental ulcers and colitis or rarely, diffuse tuberculous colitis.[14] In a study by Pulimood ***et al.***, endoscopically the distribution of macroscopic lesions was similar in the two conditions, with 60-70% of the patients showing ileocaecal involvement and about 50% showing involvement of the transverse or distal colon. Involvement of the ileocaecal valve, deformity of the caecum and stricture/stenosis were however more common in the TB patients, while fistulae were more in patients with CD.[7]

On histology, colonic TB is characterized by numerous large, well-defined granulomas, especially in the submucosa and in the granulation tissue around the ulcers, often with caseation and confluence.[7] TB granulomas are usually larger than 400 *µm* in maximum dimension with more than four sites of granulomatous inflammation per site, caseation and a band of epithelioid histiocytes in ulcer bases and location of granulomas in the caecum.[15] The granulomas in CD are fewer, smaller, never confluent or caseating and seldom found in the submucosa. In addition, there may be focally enhanced colitis, pericryptal granulomatous inflammation and architectural alteration, activity, chronic inflammation and/or deep ulceration at sites that do not show a granulomatous response in the same or adjacent segments. Although granulomas in CD are distributed throughout the colon, they are more frequent in the rectosigmoid than in TB. There is an accrual in the number of diagnoses made with an increasing number of biopsies from the rectum to the ileum.[15] Nevertheless, granulomas ***per se*** may not always be diagnostic.[16]

In a retrospective study from our institute between 1997 and 2006, we attempted to differentiate between CD and TB based on the clinical presentation, imaging and colonoscopy and followed it up with histology. Of a total of 102 patients, 60 (58.8%) were classified as TB based on clinical presentation, 20 (19.6%) as CD and 22 (21.6%) could not be differentiated based on clinical presentation, imaging and colonoscopy. Only 12 (20%) in the TB group and 13 (65%) in the CD group could be confirmed on histology. Nine (41%) patients in the group that could not be differentiated before histology could be correctly classified as TB or CD. The diagnosis changed from CD to TB in one and from TB to CD in 14. In the remaining 52 (51%) patients, the diagnosis remained elusive even at histology. (Manuscript under preparation.)

With far more pressing medical concerns for any developing country, why does this disease differentiation require a close introspection? The enormous costs of inappropriate prescription of ATT cannot be afforded by a developing country. Secondly, the potential harms of inappropriate use of ATT certainly go beyond cost concerns. Irrational use of ATT in the community has been shown to increase the chances of drug resistance among tuberculous bacteria. Also, the use of these drugs is not without adverse effects as patients develop hepatotoxicity and their safety in CD patients is not known. Also, the potential drug interactions with its adverse effects due to poly pharmacy cannot be discounted as trivial. But, in a country where TB is endemic, the chances of missing a diagnosis of TB should be balanced against inappropriate use of ATT in patients misclassified as TB instead of CD. There are still a number of unanswered questions as to whether in patients with an uncertain diagnosis, initiating a trial of ATT would be appropriate.

What are the implications of this delay in instituting treatment for CD and what are the ways by which one can be more meticulous in distinguishing these two conditions to avoid the unnecessary use of ATT? The potential problems of a delay in the treatment of CD are well known as patients with a delay in instituting treatment develop strictures requiring repeated surgeries and nonhealing fistulas with its potential nutritional consequences.

In any case of diagnostic confusion based on endoscopy, radiology and histology, the confirmatory test is to isolate the organism. Obviously, the most reliable differential method is to find evidence of ***Mycobacterium tuberculosis*** in the intestinal tissues. Unfortunately, AFB staining lacks sensitivity and specificity. In addition, the biopsy culture for ***M. tuberculosis*** is time consuming (3-8 weeks) and the results are frequently negative. It is clear that the limitation of traditional methods for differential diagnosis exist. Recently, researchers from China have highlighted the role of polymerase chain reaction (PCR) in differentiating both these conditions.[12] The most promising new approach to this problem is in the form of PCR assay, which indicated that 71.4% of intestinal TB specimens with noncaseating granulomas were positive by PCR, but no CD specimens with noncaseating granulomas amplified ***M. tuberculosis*** DNA. The study also found that 54.8% of intestinal TB specimens that tested negative for AFB tested positive by PCR assay.

The next question is that with speculations that CD is caused by ***M. paratuberculosis***,[17–20] how far is the PCR assay specific? The primers used in studies were derived from insertion sequence IS6110, which has been demonstrated to be highly specific for ***M. tuberculosis***, without interference with DNA elements from other mycobacteria, including ***M. paratuberculosis***, and to be repeated in the ***M. tuberculosis*** chromosome.[21] At present, there are no specific diagnostic tests for CD because the pathogenesis of CD is still unclear. In India, intestinal TB is more common than CD, although the numbers of patients with CD is increasing. Consequently, the diagnosis of CD in our patients remains a diagnosis of exclusion, which is only possible after excluding intestinal TB. With the advent of PCR and advanced histological techniques, the chances of misclassification have definitely decreased. Above all, it is important to remember that intestinal TB is entirely curable with early and appropriate treatment.

## CONCLUSIONS

With more and more research coming up on distinguishing TB and CD, we can expect an explosion in the current knowledge in this field. A guideline-based approach to a patient with granulomatous colitis with regard to both diagnosis and treatment would go a long way in preventing unnecessary inappropriate ATT for patients with CD and appropriate early treatment for a patient with TB. In fact, in developing countries, where TB is endemic, starting ATT would be more appropriate in cases of diagnostic confusion.
